# Volatility of the COVID-19 vaccine hesitancy: sentiment analysis conducted in Brazil

**DOI:** 10.3389/fpubh.2023.1192155

**Published:** 2023-07-07

**Authors:** Celso Machado Júnior, Daielly Melina Nassif Mantovani, Luísa Veras de Sandes-Guimarães, Maria do Carmo Romeiro, Cristiane Jaciara Furlaneto, Roberto Bazanini

**Affiliations:** ^1^Laboratory of Health Education, Institute of Innovation Multidisciplinary, Department of Administration, Municipal University of São Caetano do Sul, São Caetano do Sul, Brazil; ^2^Laboratory of Biodiversity, Biogeography and Conservation, Department Health Sciences, Institute of Biological Sciences, University Paulista, São Paulo, Brazil; ^3^Laboratory of Quantitative Methods and Informatics, Department of Administration, Institute of Analytics and Open Data, University of São Paulo, São Paulo, Brazil

**Keywords:** vaccine hesitancy, COVID-19, social media, sentiment analysis, vaccine regret

## Abstract

**Background:**

Vaccine hesitancy is a phenomenon that can interfere with the expansion of vaccination coverage and is positioned as one of the top 10 global health threats. Previous studies have explored factors that affect vaccine hesitancy, how it behaves in different locations, and the profile of individuals in which it is most present. However, few studies have analyzed the volatility of vaccine hesitancy.

**Objective:**

Identify the volatility of vaccine hesitancy manifested in social media.

**Methods:**

Twitter’s academic application programming interface was used to retrieve all tweets in Brazilian Portuguese mentioning the COVID-19 vaccine in 3 months (October 2020, June 2021, and October 2021), retrieving 1,048,576 tweets. A sentiment analysis was performed using the Orange software with the lexicon Multilingual sentiment in Portuguese.

**Results:**

The feelings associated with vaccine hesitancy were volatile within 1 month, as well as throughout the vaccination process, being positioned as a resilient phenomenon. The themes that nurture vaccine hesitancy change dynamically and swiftly and are often associated with other topics that are also affecting society.

**Conclusion:**

People that manifest the vaccine hesitancy present arguments that vary in a short period of time, what demand that government strategies to mitigate vaccine hesitancy effects be agile and counteract the expressed fear, by presenting scientific arguments.

## 1. Introduction

Vaccine hesitancy (VH) is a global phenomenon that has a major impact on population health. According to the World Health Organization (WHO), it is among the top **10** threats to global health. (1–3). The establishment of the strategic advisory group of experts (SAGE) by the World Health Organization (WHO) in 2012 was the first effort to understand, monitor, and find solutions for VH (3). Since its establishment, there has been increased interest in research related to VH (4–6), with the number of papers addressing ‘vaccine’ or ‘vaccination’ in the title increasing from 3.3% in 2019 to 8.3% in 2021 (4).

The term vaccine hesitancy is still under development and is subject to controversy. The initial proposal from the World Health Organization (WHO) indicates VH as a behavioral phenomenon, where individuals neither accept nor completely reject the possibility of vaccination (7, 8). The divergence that is established in the definition of the term VH lies in the understanding that it is not a social behavior, but rather “a state of indecisiveness regarding the decision to become vaccinated” (3), a position shared by other researchers (9).

Vaccine hesitancy is a global phenomenon. Still, public acceptance of the vaccine varies depending on the region (10). Countries in Asia have higher rates of vaccine acceptance (Malaysia with an acceptance of 94.3%, Indonesia of 93.3%, and China of 91.3%) compared to Europe (France with an acceptance of 58.9%, Poland of 56.3%, and Italy of 53.7%) and the United States (acceptance of 56.9%), which have lower levels of vaccine acceptance (11).

VH is positioned as a complex and dynamic social phenomenon, for which the influencing factors are still being researched (9, 11). Sex, educational level, age, geographical location, income, professional occupation, race and ethnicity (12), living with five or more people (13), pregnancy (14), being a health professional, and having previous vaccination experience (15) are among the factors that influence VH. Research studies establish other factors, such as group and individual aspects that involve a lower understanding of the risk, lower fear of contamination, believing that the disease is not serious, and not having heart diseases, as influencing factors of VH (16). The concepts of cognition or affection, behavior, and decision making have also been related to VH (3), which is also identified as linked to government credibility, incorporating aspects related to wanting more data for decision making, doubts about vaccine efficacy and safety, feeling that personal rights are being infringed upon, and lack of trust in the government and health care institutions (14, 15).

The factors influencing VH are proposed from two constructs: the HBM (12) and the 7C (17). The HBM construct examines the influence of perceived susceptibility, perceived severity, perceived benefits, perceived barriers, cues to action, and self-efficacy on VH (18), which are mostly conditional on the prevalence of demographic, psychosocial, and structural variables (12). While the 7C construct points to trust, complacency, convenience (or constraints), risk calculation, collective responsibility, compliance, and conspiracy as factors driving VH (9, 17, 19).

The VH in Brazil is a phenomenon that originated before the COVID-19 pandemic and evolved from 2016 (20). It is possible to identify this decline in vaccination coverage in the analysis of mandatory vaccination for Human Papillomavirus (HPV) among girls aged 9 to 14 years, which showed a vaccination coverage rate of 83.4% for the first dose and only 55 0.6% in the second dose. For the general Brazilian vaccination system in 2021, the coverage rate considering all vaccines made available by the public network was 59% of citizens (21). In Brazil, there are studies that show significant variability of information about the population’s VH. Among the studies that analyzed VH in Brazil, one stands out, which consolidates the analysis of eleven other studies on the subject, in which a variation from 8.2 to 34% of vaccine hesitancy for COVID-19 was found (22), the consolidation of these studies, indicated an average vaccine hesitancy of 11.1%. A justification for the discrepancy identified in these studies is based on the large territorial extension of the country, with locations that present different behaviors in relation to the general average of the country. In this sense, research carried out in the Brazilian state of Maranhão identified a rate of hesitation to vaccinate against the SARS-CoV-2 virus of 17.5% (23), which is higher in comparison with an average of 11.1% (22).

Overall, previous studies have addressed the factors influencing VH and its variation across locations. However, it is unclear whether VH is subject to the volatility of people’s decision to take the vaccine, as public sentiment is volatile (24), or if the influence of digital media platforms on the decision to take the vaccine (25). Therefore, the objective of this study was to identify the variability of vaccine hesitancy manifested in social media. Overall, based on the papers reviewed, this is one of the first studies to explore the volatility of VH using social media as a data source, a context that meets the view that public sentiment tends to be volatile toward VH (24). It is also worth noting, the existence of studies that, relying on Twitter databases, have analyzed people’s sentiment toward COVID-19 (26–28) and toward the COVID-19 vaccine (29–31).

## 2. Materials and methods

### 2.1. Data extraction and pre-processing

A dataset was obtained from Twitter’s Application Programming Interface (API). The data search was divided into three periods delimited by the months of October 2020, June 2021, and October 2021, and encompassed Brazilian data. The months of data collection were defined to identify different moments of the vaccination process for COVID-19. Additionally, each month’s data was divided into one-week periods, with October 2020 and June 2021 having five analysis periods, and October 2021 having four. The keywords used to search the tweets were “COVID-19,” “vaccine” and “vaccination” in Brazilian Portuguese. This data collection technique has been observed in similar studies (26, 27, 31–33). Opted to use Brazilian Portuguese, as the research is being carried out by a group of Brazilian researchers, who seek to identify mechanisms for predicting future pandemics. The capture of the text, only in Brazilian Portuguese, excluding tweets generated in Portuguese from other countries, as well as the use only of tweets generated from the Brazilian territory, was possible using the knowledge-based model, which captures a wide variety of locative references from tweets, like proposed by Martínez and Pascual (34). It is worth mentioning, that the authors of this article make a collaborative effort to expand knowledge on the subject, approaching productive research groups that address the theme of vaccine hesitancy and coping with COVID-19 (35–40). Thus, the findings of this research make up a contribution to the mosaic of international studies that address VH, however, caveats must be taken when generalizing their findings, because they are characteristic of the Brazilian population.

The exported data had issues with character encoding (accents and other special formats) and provided a date formatting presented as year, day, month, hour (including minutes and seconds) that is different from the Brazilian standards. To correct these issues, it was necessary to use the R software to help edit the data, since the database had over 1 million observations. During data extraction, were removed all duplicate tweets and the same tweet from the same user. After this step, the data were exported from R as a .csv file, generating one file per month analyzed. It is worth mentioning, that in this study, there are no independent or dependent variables, since the paper focused on text sentiment analysis.

The .csv data was imported into the Orange Data Mining software to conduct the sentiment analysis, an unsupervised text mining technique (without an initial reference/classification as the initial input into the software) that aims to extract the emotion present in a message.

In the Orange software, the text corpus was pre-processed to remove stop words (prepositions, pronouns, etc.). Only whole words without numbers or other characters (Regexp) are maintained. Specific features of the Twitter language (for example, the use of hashtag and at signs) are identified and URLs removed from the text.

### 2.2. Sentiment analysis

The sentiment analysis was performed with the help of the software Orange, in which a sentiment analysis was initially performed with the Multilingual sentiment lexicon in Brazilian Portuguese, which contains a previous mapping of words categorized as positive and negative. It is noteworthy, that the research addresses the feelings of the Brazilian population, so the generalization of its findings must be carried out with reservations. In this method, each word is categorized as negative, positive, or neutral and the overall sentiment score of the text is calculated (positive words-negative words/total words). Each tweet was assigned a score for sentiment that ranged from −1 to 1, where a classification above 0 = positive, below 0 = negative, and equal to 0 = neutral. Since the article is specifically focused on vaccine hesitancy, the analysis presented below involved the manual selection of a few tweets that showed adherence to the topic, according to the definitions presented in the literature. Due to the size (over 1 million tweets) and subject of the database, it would be impossible to perform this analysis automatically, using only the tweets related to vaccine hesitancy. The approach adopted presents similarities with studies on this subject (29, 30, 41, 42).

## 3. Results

The sentiment analysis of the VH was performed in three different periods of the vaccination process, which are presented in charts divided into 1-week periods. This distribution was planned to observe the sentiment of VH in short periods (week) within the same month, and in longer intervals (over 4 months). The analysis relied on the selection of 1.047.018 tweets adhering to the topic of vaccine hesitancy. However, the overall sentiment of the analyzed tweets for October 2020 was −0.0438, for June 2021 was −0.0510, and for October 2021 was −0.0519. The overall sentiment was therefore slightly negative in all periods.

In October 2020, the availability of a vaccine for COVID-19 was identified in the Brazilian media. Three vaccine possibilities were discussed, those offered by Pfizer/Biontech, Oxford/AstraZeneca, and Sinovac/Butantan (CoronaVac). Subsequently other possible vaccines to be purchased by Brazil were identified. However, only these three vaccines were employed in greater volume. The sentiment analysis was developed initially by the researchers’ perspective (presented in the sentiment analysis performed) and then by the score provided by the Orange Software (sentiment score column). The purpose of this double analysis was to identify the alignment of the researchers’ perception with that obtained later in the analysis developed in the Orange software. From this perspective, some differences in positioning were identified between the analyses, however, without contrasts (variation between negative and positive, as the divergences were centered on the neutral sentiment). For this research, each divergence was analyzed individually, and used to deepen the understanding of the data and broaden the discussion. Table 1 presents the sentiment analysis for October 2020. It is noteworthy that the sentiment analysis is focused on VH and not on other aspects pointed out in the tweets. The sentences that exemplify the sentiment identified and presented in the first columns of the tables were translated from Portuguese to English to present the research by the authors.

**Table 1:** Sentiment analysis of the COVID-19 vaccine in October 2020.

	Tweet example	Sentiment	Sentiment Score
1	“In a time of pandemic, confinement is a stimulus for creativity. It is worth remembering that it was in isolation from the bubonic plague that Isaac Newton created the Theory of Gravity.”	The messages address the topic. There are no factors related to the vaccine yet. There is no discussion on the name of the vaccine (Neutral sentiment).	−0.296 (negative)
2	“With the pandemic one must have the Mandalorian mindset – never take off your mask.” (Mandalorian is a character from a series based on the Star Wars movie, who constantly wears a mask).	The vaccine is still not presented as a possibility, the main focus of people’s attention is related to preventive measures, such as using masks (Neutral sentiment).	0.0 (neutral)
	“The pandemic will kill many from depression because of social isolation. Human beings need to socialize, to hug, to talk.”	Expression of concern for the psychological state of the people, and a sign of saturation of the lockdown model (Neutral sentiment).	0.0 (neutral)
3	“The COVID-19 pandemic and social isolation have profoundly changed the routine of Brazilians, affecting family and work relationships, and healthcare as well.”	Expression of concern for the psychological aspects of people, and a sign of saturation of the lockdown model (Neutral sentiment).	0.0 (neutral)
	“I would really like my student side to become active again. I have things to study and I cannot concentrate.”	The interference of social distancing, in people’s lives, with an impact on professional and educational activities (Neutral sentiment).	0.0 (neutral)
4	“Except for the Russian vaccine, all are following testing protocols and developmental steps.”	Relevant moment, because it marks the beginning of the discussion about the vaccines that will be used in Brazil, as well as the approval process, with two possibilities appearing with greater intensity: (i) AstraZeneca (Oxford) and (ii) CoronaVac (Butantan). (Positive sentiment).	0.34 (positive)
5	“But the biggest impact to the country could be with the amount of people who suffer from side effects worse than the virus itself. There have been no animal tests on the recommended scale.” “[It is] absurd to force a vaccine with no proven efficacy…”	The messages complain about the mandatory vaccination against COVID-19, especially with a vaccine supposedly without scientific proof. Most of the messages are against the obligation (Negative sentiment).	0.0 (neutral)

Source: research data (2023).

The data show the onset of vaccine topic within the spectrum of solutions for the COVID-19 pandemic in Brazil. In the first insertion the VH phenomenon is already observed, focusing on the efficacy of the vaccines that are being proposed. The data show an alternation between negative, neutral, and positive sentiments; however, with a prevailing neutral positioning.

According to the Brazilian government priority planning, COVID-19 vaccines began to be delivered in January 2021 through prioritizing health care workers and the older adults. By June 2021, most of the population identified as priority population had already received the first dose of the COVID-19 vaccine. Thus, the vaccination process was already within reach of the population, however, still following the planned schedule of making the vaccine available in an order of decreasing age. Table 2 presents the sentiment analysis in June 2021.

**Table 2:** Sentiment analysis of the COVID-19 vaccine in June 2021.

	Tweet example	Sentiment	Sentiment Score
1	“Worst thing. People are suffering from vaccine backlash.”	The central focus of the tweets was the manifestation of concerns with possible reactions from the vaccines, especially those originating from the AstraZeneca vaccine. In general, they were satisfied with the immunization despite the symptoms (Neutral sentiment).	0.0 (neutral)
	“Have any pregnant women been vaccinated against COVID and had a reaction? I am scared to death.”	Concern about the effect of the vaccine on women who are pregnant (Negative sentiment).	0.0 (neutral)
2	“Wow, who would have thought that mass vaccination would be the solution, right? Nobody saw that coming.”	The manifestations express the vaccine as the solution to the pandemic (Positive sentiment).	0.0 (neutral)
	“A CoronaVac study in Serrana shows that the pandemic can be controlled.”	Great interest in the CoronaVac vaccine test results from the town of Serrana in the countryside of the state of São Paulo (Brazil) (Positive sentiment).	0.0 (neutral)
	“You have to see which is the vaccine, because they showed one on TV that was suspended for pregnant women because it was giving a reaction.”	Concern about the effect of the vaccine on women who are pregnant. Perception that the vaccine manufacturer can influence the outcome (Negative sentiment).	0.0 (neutral)
3	“City Hall just released the return of Pfizer’s second dose vaccination.”	The population’s attention is on the Pfizer vaccine. The other options do not appear, indicating a preference of the population for Pfizer (Neutral sentiment).	0.0 (neutral)
	“Vaccination of players and games without fans are among the country’s requests to host the Copa America.”	Although not directly related to the vaccine, the topic of the America’s Cup (South American soccer tournament) appears prominently, with athletes’ immunization in the background (Neutral sentiment).	−0.296 (negative)
	“If you are pregnant, I do not know if it is good to take the vaccine.”	Concern about the effect of the vaccine on women who are pregnant (Negative sentiment).	0.0 (neutral)
4	“The problem is not the America’s Cup, but who authorized it. As everything in this pandemic is political, I believe that the media has a share of the blame in the uncontrolled pandemic.	The tweets address the Brazilian government’s decision to host the Copa America, refused in other countries. The tweets indicate the importance of players being vaccinated (Neutral sentiment).	−0.435 (negative)
	“All people involved in the America’s Cup are vaccinated with the two doses of the vaccine”		0.0 (neutral)
	“Find out where there is Pfizer vaccine for pregnant women.”	Concern about the effect of the vaccine on women who are pregnant. Perception that the vaccine manufacturer can influence the outcome (Negative sentiment).	0.0 (neutral)
	“Very nice, coercing teachers and education professionals to receive the vaccine in exchange to being forced to go back to work.”	With the return of face-to-face classes, teachers are speaking out against making the vaccine mandatory. (Negative sentiment).	−0.373 (negative)
	“Forcing teachers to come back face-to-face, to have to take the vaccine.”		0.0 (neutral)
5	“I am so happy when I see people close to me taking the vaccine, even without being a friend. I love it so much!!!”	Enthusiasm for the positive result of the vaccine on the spread of COVID-19, as well as for the reduction in the number of deaths (Positive sentiment).	0.202 (positive)
	“How good it is to lay your head on the pillow knowing that my parents, my sister and I have taken the first dose of the vaccine against COVID-19.”	The focus of the messages is no longer on the vaccine brand, but on having taken the first dose. (Positive sentiment).	0.296 (positive)
	“Teachers criticize the obligations for face-to-face return, such as the vaccination requirement.”	With the return of face-to-face classes, teachers are demonstrating against the obligation to take the vaccine. (Negative sentiment).	0.0 (neutral)
	“Here in Fortaleza, teachers are being blackmailed into taking the vaccine and signing a term to return to classes in August.”		0.0 (neutral)

Source: research data (2023).

The data reflect an intense period of the vaccination process, with most of the tweets addressing this topic. The sentiment expressed is positive and adherent to the vaccination process promoted by the Brazilian government. The tweets present three interesting features and approaches, despite dealing with a common trend, which are: (i) the positive result of the CoronaVac vaccine (Sinovac/Butantan) tests carried out in the city of Serrana is expressed as a positive sentiment; (ii) despite the positive result of the CoronaVac vaccine, the population preferred the Pfizer/Biontech vaccine; (iii) people are satisfied for having taken the first dose of the COVID-19 vaccine and being close to taking the second dose. These analyzed aspects are intertwined in the understanding of the benefits resulting from taking the vaccine. However, two specific groups with VH are identified, the pregnant women and the teachers in the education network. The hesitation of pregnant women permeates the concern of the vaccines affecting the pregnancy or the fetus. In this context, there are discussions as to which vaccine is the safest. In the case of teachers, it is worth noting that classes were paralyzed in Brazil in March 2020, when classroom activities were expected to return in August 2021, causing concern among teachers. In this approach, it is possible to have a double interpretation of the data. The first one is related to the VH, while the second is related to the hesitation of returning to the classroom, regardless of having taken the vaccine. However, under both terms the vaccine is positioned as an element of argumentation for the hesitation expressed by the teachers. Table 3 presents the sentiment analysis for October 2021.

**Table 3:** Sentiment analysis of the COVID-19 vaccine in October 2021.

	Tweet example	Sentiment	Sentiment score
1	“Oh my God, if it works out now, I’m going to get a vaccine.”	The messages focus on vaccination, which is identified as something positive (Positive sentiment).	1.0 (positive)
	“Everyone who wants a vaccine takes it.”	With vaccine availability for all, the manifestation of individuals not wanting to get vaccinated against COVID-19 is observed (Negative sentiment).	0.0 (neutral)
	“I do not agree with the minister. Vaccinate whoever wants to. I will not be a guinea pig.”		0.0 (neutral)
2	“With the vaccine for everyone now, is the second dose guaranteed for those who have already taken the first?”	Cycle of concern for the second dose of vaccination (Positive sentiment).	0.0 (neutral)
	“It would be ridiculous to force people to get a vaccine at an experimental stage.”	The concern is related to the developmental stage of the vaccine, as well as to the need for approval of the vaccine by the regulatory agency in Brazil (Negative sentiment).	0.0 (neutral)
	“[someone] badly wanted to vaccinate people without the consent of ANVISA.”		−0.526 (negative)
3	“YES! Wow, I miss that. Waiting for the pandemic to end so I can come back.”	The vaccine does not appear as the prevailing theme, but rather the process of ending the pandemic, i.e., the post-pandemic (Neutral sentiment).	0.0 (neutral)
	“Now they are going to force everyone to take this crap of which the long-term effects are unknown.”	The uncertainty is related to the long-term effect of the vaccines on people, i.e., what possible adverse effects could occur in the future (Negative sentiment).	−0.700 (negative)
	“None of the vaccines they are giving to the people have INSTRUCTIONS informing the reactions.”		−0.373 (negative)
4	“I’m glad the pandemic is over. I simply could not stand this social detachment any longer.”	The perception is that the pandemic is over. No messages are identified pointing to the collaboration of the vaccine in this process. (Neutral sentiment).	0.0 (neutral)
	“They made me take the vaccine. Imagine my regret. I should not have taken it.”		0.0 (neutral)
	“They’re not putting a gun to anyone’s head to force them to take this garbage, so those who choose to get it are the ones to blame.”	Statements against the decision of having received the vaccine (Negative sentiment).	0.0 (neutral)

Source: research data (2023).

The data for October 2021 portray a period of a more advanced stage of the population vaccination process. This period showed a greater VH manifestation, probably due to the availability of the vaccine for everyone, encouraging those who are more resistant to the vaccine to manifest with greater intensity. The data show an alternation between negative and neutral sentiments, with little occurrence of positive sentiments. This indicates that almost a year after the beginning of the vaccination process VH still exists, consisting of a resilient phenomenon that, therefore, should be the object of planning and actions aimed at its mitigation. The tweets also indicate the expectation for the end of the COVID-19 pandemic. In this perspective, the vaccination process, added to other factors such as the reduction of infection, reduction of deaths, desire to return to normal activities, with the potential interaction among these factors, have set an important milestone for people to interpret the period as the end of the pandemic. It is worth mentioning, that these characteristics observed in the tweets, as well as the brands of vaccines available, the period and priority of vaccination of the population, are related to characteristics of the vaccination process in Brazil, thus presenting intrinsic limitations of generalization of the findings of this research.

## 4. Discussion

Previous studies have revealed that vaccine hesitancy (VH) is a dynamic phenomenon influenced by many variables that impact it in a positive or negative way (9, 11). The variables that influence VH operate differently in each region depending on characteristics of the society (10, 11). Residents of sub-Saharan Africa, for example, feared being victims of vaccine experimentation (43).

Vaccine hesitancy research studies show variations in vaccine acceptance rates for COVID-19 (10, 11, 44). Note that VH is not a behavior, but rather a state of indecision (3, 9), thus subject to temporal factors (45) and changes resulting from the dissatisfaction or satisfaction of society concerning access to information and actions of the government (44). The identification of factors that collaborate with or mitigate VH stand out for enabling the expansion of vaccination coverage, and thus facilitating the achievement of the theoretical 90% or higher rate needed to establish collective immunity (46).

The first set of data analyzed shows the period before the vaccination process began (October 2020). The data indicate that this period was characterized by an initial phase of indifference to vaccine-related aspects and greater attention to the protocols adopted by society. At the end of this period, the manifestations of feelings related to the vaccine, both positive and negative, can be observed. Since it is a new vaccine, the reservations are related to the adverse effects resulting from its use and to its efficacy. The identified resistance occurs in the context of a new product, for which little is known concerning efficiency and potential adverse effects (11, 15, 16, 47). These initial manifestations express concerns with the source of the vaccine, i.e., with which laboratory is developing it, highlighting the need for the government to establish partnerships with research and production laboratories to provide as much information as possible. Subsequent evidence showed that some vaccines were imperfect or even had a declining protection index, thus favoring new COVID-19 outbreaks (48). In Brazil some politicians assured that the vaccine made contamination impossible, which increased VH when the first cases of contamination by COVID-19 of vaccinated people surfaced. From this perspective, the lack of trust in the government increased VH (1, 10, 14, 15, 25, 49). The inappropriate manifestation of politicians regarding the vaccine strengthens VH, impacting trust in the government and in the COVID-19 vaccine. This suggests that politicians should base their communications on evidence provided by the experts and not on their own feelings about vaccination.

The second block of data analyzed concerns the month of June 2021, which is marked by a significant portion of the Brazilian population having already received at least the first dose of the vaccine. Meanwhile, VH was expressed as negative sentiments toward the vaccine. In this interval, three phenomena associated with the VH in relation to the COVID-19 vaccine stood out: pregnant women, the Copa América being held in Brazil (soccer championship between the national teams of South American countries), and the return to school.

The suitability of the COVID-19 vaccine for pregnant women was a dominant theme throughout the period. VH was expressed in questioning the suitability of vaccines in general as well as the suitability of one of the vaccines available (50). However, in both VH motivations, it is possible to ponder that doubting the suitability of the vaccine for pregnant women may cause this negative sentiment toward the vaccine to overflow to the society.

Another aspect that pervaded the tweets in the analyzed period was the Copa América being held in Brazil. In this case, a negative aspect regarding the event was observed, as the risks and appropriateness of holding the tournament were questioned. From this perspective, the manifestation of HV was not identified. On the contrary, a concern for the vaccination of the tournament players was observed. Thus, the calculation of contamination risk and collective responsibility acted as elements of positive reinforcement for vaccination (17, 51). Additionally, the attention given to sport-related activities during a pandemic outbreak can positively influence social connections and relieve psychological stress (52).

Another interesting aspect observed in this period involves primary and secondary school teachers. Some teachers interpreted the need to get vaccinated as a coercion to return to classroom activities in schools. From this perspective, VH is not explicitly perceived. However, the teachers’ fear of returning to the classroom heightened VH in general. We can thus infer that their professional activity is influencing the vaccination process (12, 15), and causing the feeling that their personal rights are being infringed upon (15). In Brazil, primary and secondary education is largely the responsibility of municipal and state governments, which halted school classes in 2020. In turn, studies indicate that students in schools, with greater confidence in the management of the pandemic, tend to be more willing to be vaccinated (53). This block of analysis (June 2021) shows the possibility of observing multiple social factors interfering with VH at short time intervals (1 month).

This period is also a turning point as the pandemic is concerned, namely the proximity of the end of the crisis. At the end of June 2021, the beginning of manifestations alluding to the approach of the end of the COVID-19 pandemic are identified, and most of them relate this event to the COVID-19 vaccination process, i.e., there is a positive feeling toward vaccination and its influence on the end of the crisis.

The third block of analyzed data includes tweets from October 2021. In this period a significant portion of the population had already been vaccinated, and in many cases including the second dose. Given the advanced stage of vaccination, the COVID-19 vaccine was already available to the entire population. Until then, vaccination followed a decreasing schedule in relation to people’s age, so the older adults had priority. In this perspective, we have the VH manifestations resulting from vaccine availability (14). Such availability caused those who were hesitant to manifest their opinion with greater intensity. Despite the existence of COVID-19 variants circulating in Brazil, we did not encounter any manifestation of VH related to this issue (48).

An interesting fact is that the increased occurrence of HIV manifestations in the period when vaccination is completed may be associated with the occurrence of reinfection and shorter duration of the immunity provided by the vaccine, which contradicts the initial information provided by politicians to the population (54). Other plausible explanations for this increase are the prohibition of access of those who did not get the vaccine to certain public places and the political discussion of establishing a mandatory vaccination “passport” for the population. These actions highlight the association of VH with government policies (1, 14, 15, 24), with the efficiency and potential adverse effects of the vaccine (11, 14, 15, 47), and, in many cases, with the short time of vaccine development (16). In this period, one can also observe the manifestation of regret for having taken the vaccine. This characteristic establishes an important point of future investigation, as it constitutes a new attitude of people that differs from hesitation before the fact – the “vaccine regret” (VR). The VR can affect the VH and drive people to avoid getting booster doses.

It is worth mentioning, that no studies were identified in the literature that presented the concept of VR. Thus, this study establishes a new perspective to be analyzed in future studies. However, when performing a search in the literature, the term “anticipated regret” was identified (55, 56). Anticipated regret is related to the feeling that we may regret a decision in the future, and in this condition, it’s can potentially be used as a predictor of vaccine behavior (57).

Typically, studies of anticipated regret are related to anticipated regret in decisions to vaccinate against Human Papillomavirus (HPV) (58, 59) and address the antagonism between the daughter’s anticipated regret of becoming more sexually active after vaccination and regret by inaction if the daughter develops an HPV infection if she is not vaccinated (60). However, this concept was also used in a study of vaccination against influenza (61) and COVID-19 (58).

The joint analysis of the three researched periods made it possible to identify that the VH manifests itself continuously and in association with themes that are affecting society, thus characterizing it as a social and dynamic phenomenon (9, 10). In manifestations expressed in the social media, the VH was associated with vaccine manufacturers and related to the public sentiment (9, 24). Thus, social media is positioned as an important channel for to manifest VH (10, 25). Therefore, it should also be a resource used by the government to disseminate information that reduces VH. The VH is volatile on social media, as it appears related to various social topics that change very quickly. Thus, the government’s action on social media to reduce VH must be agile so as not to distance itself from the topics that are being addressed. It is important to emphasize, that if the government does not quickly combat tweets that encourage vaccine hesitancy, these messages can lead to an adverse result in the vaccination process, regardless of the frequency of communication (52). This perspective validates VH as a social process under the influence of multiple meanings and logics, circumscribed to the individual and collectivity.

Agile government efforts may prevent occurrences such as the one observed in France, where a vaccine uptake level that positively fluctuated between 62.0 and 77.1% in March and April 2021 reduced to a markedly lower level of 58.9% in June 2021, likely due to concerns related to vaccine safety and efficacy (54, 62, 63). Notably, the potential positive association between trust in the government and COVID-19 vaccination involves sociopolitical factors related to public health policy implementation (64).

In vaccine hesitancy analyses, it becomes relevant to incorporate the position of healthcare workers (HCW) in the vaccination process. The inclusion of HCW in the effort to reduce VH comes from the fact that people trust these professionals and follow their guidelines (65–67). The influence of HCW guidance on reducing VH is significant (68) and proved to be important when coping with the COVID-19 pandemic. A study carried out in June 2020, with the population that presented VH for COVID-19, indicated that 51.9% presented VH even after the indication of HCW vaccination (68). The same study was carried out in June 2021, and it was identified that this value had reduced to 35.8% (68), that is, during the pandemic the population increased its confidence in health professionals, and in their recommendations for the vaccination.

The influence of the HCW, for the reduction of VH, with the population, can be compromised if these professionals also present this hesitation. There is no consensus on the dimension of VH in HCW, however two surveys stand out. The global survey, which analyzed 35 studies in the area, indicated a variation between 4.3 and 72% of VH in HCW, depending on the country in which they operated (69). Another survey, which analyzed HCW according to their training, identified an average of 8.1% of VH among HCW, with the categories with the lowest VH indices being Physicians (3.1%) and Nurses (6.5%) (68). In this perspective, it is worth noting that the HCW also have VH, however, they do not express their concerns due to institutional and social pressures (70). Thus, the unspoken vaccine hesitancy of HCW is identified as an element of attention of health systems, as it can influence the reduction of confidence in the vaccine by the public.

Two relevant aspects of the participation of HCWs in the HV mitigation process are thus identified: the first is that they influence people’s decision to take the vaccine, so this aspect should be used in favor of awareness campaigns. The second aspect indicates that HCW may have unspoken vaccine hesitancy, which requires health system managers to establish awareness programs, with this specific public, that include autonomy and freedom to express their hesitations.

The attention devoted to COVID-19 VH research should support ongoing efforts begun by the WHO in 2012 (3), aimed at defining, monitoring, and mitigating VH. The benefit of the cooling of the COVID-19 pandemic should not impact the importance of VH research, as there are other infections that spread in waves, affecting all of society. One example is the influenza virus responsible for infecting 1 billion people per year (48, 71), resulting in 290,000 to 650,000 influenza-related respiratory deaths (72).

## 5. Conclusion

This study defines vaccine hesitancy as a complex and dynamic social phenomenon, subject to several influencing factors. Among the considerations and findings of this research are the arguments presented by society for not getting vaccinated, which vary over short periods of time, surely within the period of a month. Thus, actions aimed at reducing vaccine hesitancy must be agile in capturing the concerns that permeate society, and in presenting information that encourages people to take the vaccine. In Brazil, vaccine hesitancy studies are still in the initial phase, so this research presents itself as a strength, for Brazilian society, which has its own cultural traits. This characteristic establishes limitations, when applying the knowledge developed in this study, in countries with cultures other than Brazilian.

### 5.1. Limitations

The origin of the data did not allow raising variables common to other studies, such as: age, sex, education, professional occupation, race and ethnicity. In this perspective, this study presents additional information on vaccine hesitancy. Additionally, the data from this study refer to the Brazilian population, so caveats should be established in generalizing the findings of this research to other populations.

## Data availability statement

The datasets presented in this study can be found in online repositories. The names of the repository/repositories and accession number(s) can be found below: https://docs.google.com/spreadsheets/d/1getfuzuxOFc_4V_TT_vm2TZHrxe3efUK/edit?usp=share_link&ouid=117126578285990502154&rtpof=true&sd=true.

## Ethics statement

Ethical review and approval were not required for the study of human participants in accordance with local legislation and institutional requirements. Data were collected from social media preserving anonymity.

## Author contributions

CM, DM, LS-G, MR, CF, and RB were involved in the analysis and interpretation of the data. CM, DM, and LS-G did the drafting of the article. CM and LS-G performed the statistical analysis. DM, MR, CM, and RB improved the quality of the English of the manuscript. CF wrote the final version all the manuscript. All authors revisited critically the manuscript for intellectual content, approved the version to be published and agree to be responsible for all aspects of the work, and involved in the conception and design of the study.

## Funding

This work was funded by a Research Incentive Scholarship offered by the University of São Paulo – USP.

## Conflict of interest

The authors declare that the research was conducted in the absence of any commercial or financial relationships that could be construed as a potential conflict of interest.

## Publisher’s note

All claims expressed in this article are solely those of the authors and do not necessarily represent those of their affiliated organizations, or those of the publisher, the editors and the reviewers. Any product that may be evaluated in this article, or claim that may be made by its manufacturer, is not guaranteed or endorsed by the publisher.

## References

[1] Nuwarda RF Ramzan I Weekes L Kayser V. Vaccine hesitancy: contemporary issues and historical background. Vaccine. (2022) 10:e1595. doi: 10.3390/vaccines10101595, PMID: 36298459 PMC9612044

[2] Larson HJ. Defining and measuring vaccine hesitancy. Nat Human Behav. (2022) 6:1609–10. doi: 10.1038/s41562-022-01484-7, PMID: 36418535 PMC9684976

[3] Bussink-Voorend D Hautvast JL Vandeberg L Visser O Hulscher ME. A systematic literature review to clarify the concept of vaccine hesitancy. Nat Hum Behav. (2022) 6:1634–48. doi: 10.1038/s41562-022-01431-6 35995837

[4] Attwell K Hannah A Leask J. COVID-19: talk of ‘vaccine hesitancy’ lets governments off the hook. Nature. (2022) 602:574–7. doi: 10.1038/d41586-022-00495-8, PMID: 35194212

[5] Calac AJ Haupt MR Li Z Mackey T. Spread of COVID-19 vaccine misinformation in the ninth inning: retrospective observational infodemic study. Jmir Infodemiol. (2022) 2:e33587. doi: 10.2196/33587, PMID: 35320982 PMC8931848

[6] Hartert TV Ortiz JR. Need for improved global measurement of early childhood respiratory syncytial virus disease. Lancet. (2022) 399:1993–5. doi: 10.1016/S0140-6736(22)00623-7 35598609

[7] Fisher KA Bloomstone SJ Walder J Crawford S Fouayzi H Mazor KM. Attitudes toward a potential SARS-CoV-2 vaccine: a survey of U.S. adults. Ann Intern Med. (2020) 173:964–73. doi: 10.7326/M20-3569, PMID: 32886525 PMC7505019

[8] World Health Organization. Report of the SAGE working group on vaccine hesitancy. (2014). Available at: (https://www.who.int/immunization/sage/meetings/2014/october/1_Report_WORKING_GROUP_vaccine_hesitancy_!nal.pdf).

[9] Wiysonge CS Ndwandwe D Ryan J Jaca A Batouré O Anya BPM. Vaccine hesitancy in the era of COVID-19: could lessons from the past help in divining the future? Hum Vaccin Immunother. (2022) 18:1–2. doi: 10.1080/21645515.2021.1893062, PMID: 33684019 PMC8920215

[10] Karafillakis E Van Damme P Hendrickx G Larson HJ. COVID-19 in Europe: new challenges for addressing vaccine hesitancy. Lancet. (2022) 399:699–701. doi: 10.1016/S0140-6736(22)00150-7, PMID: 35123665 PMC8813064

[11] Sallam M. COVID-19 vaccine hesitancy worldwide: a concise systematic review of vaccine acceptance rates. Vaccine. (2021) 9:1–14. doi: 10.3390/vaccines9020160 PMC7920465 33669441

[12] Limbu YB Gautam RK Pham L. The health belief model applied to COVID-19 vaccine hesitancy: a systematic review. Vaccine. (2022) 10:1–13. doi: 10.3390/vaccines10060973, PMID: 35746581 PMC9227551

[13] Fajar JK Sallam M Soegiarto G Sugiri YJ Anshory M Wulandari L. Global prevalence and potential influencing factors of COVID-19 vaccination hesitancy: a meta-analysis. Vaccine. (2022) 10:1–20. doi: 10.3390/vaccines10081356 PMC9412456 36016242

[14] Truong J Bakshi S Wasim A Ahmad M Majid U. What factors promote vaccine hesitancy or acceptance during pandemics? A systematic review and thematic analysis. Health Promot Int. (2022) 37:daab105. doi: 10.1093/heapro/daab105 34244738

[15] Peterson CJ Lee B Nugent K. COVID-19 vaccination hesitancy among healthcare workers - a review. Vaccine. (2022) 10:1–30. doi: 10.3390/vaccines10060948 PMC9227837 35746556

[16] Aw J Seng JJB Seah SSY Low LL. COVID-19 vaccine hesitancy - a scoping review of literature in high-income countries. Vaccine. (2021) 9:1–21. doi: 10.3390/vaccines9080900, PMID: 34452026 PMC8402587

[17] Geiger M Rees F Lilleholt L Santana AP Zettler I Wilhelm O. Measuring the 7Cs of vaccination readiness. Eur J Psychol Assess. (2022) 38:261–9. doi: 10.1027/1015-5759/a000663

[18] Rosenstock IM. Historical origins of the health belief model. Health Educ Monogr. (1974) 2:328–35. doi: 10.1177/109019817400200403 299611

[19] Betsch C Schmid P Heinemeier D Korn L Holtmann C Böhm R. Beyond confidence: development of a measure assessing the 5C psychological antecedents of vaccination. PLoS One. (2018) 13:e0208601. doi: 10.1371/journal.pone.0208601, PMID: 30532274 PMC6285469

[20] Sociedade Brasileira de Imunizantes (SBIm). Especialistas se reúnem para debater o fenômeno da hesitação vacinal no Brasil. (2021). Available at: (https://sbim.org.br/noticias/1619-especialistas-se-reunem-para-debater-o-fenomeno-da-hesitacao-vacinal-no-brasil).

[21] de Fortaleza Prefeitura. I Fórum Hesitação em Vacinar alerta sobre a importância da participação da sociedade nas campanhas de imunização. (2023). Available at: https://www.fortaleza.ce.gov.br/noticias/i-forum-hesitacao-em-vacinar-alerta-sobre-a-importancia-da-participacao-da-sociedade-nas-campanhas-de-imunizacao (Accessed May 13, 2023).

[22] Leite ESF Martins MG Martins CMDCR. Hesitação vacinal e seus fatores associados no contexto da pandemia de COVID-19 no Brasil. Cadernos Prospecção. (2023) 16:484–502. doi: 10.9771/cp.v16i2.50880

[23] Oliveira BLCAD Campos MAG Queiroz RCDS Alves MTSSDB Souza BFD Santos AMD. Prevalência e fatores associados à hesitação vacinal contra a COVID-19 no Maranhão. Brasil Revist Saúde Pública. (2021) 55:1:12. doi: 10.11606/s1518-8787.2021055003417, PMID: 33909868 PMC8046425

[24] Pertwee E Simas C Larson HJ. An epidemic of uncertainty: rumors, conspiracy theories and vaccine hesitancy. Nat Med. (2022) 28:456–9. doi: 10.1038/s41591-022-01728-z, PMID: 35273403

[25] Larson HJ Murray CJ GE. The vaccine-hesitant moment. N Engl J Med. (2022) 387:58–65. doi: 10.1056/NEJMra2106441, PMID: 35767527 PMC9258752

[26] Dubey AD. Twitter sentiment analysis during COVID-19 outbreak. (2020). Available at: https://papers.ssrn.com/sol3/papers.cfm?abstract_id=3572023

[27] Priyadarshini I Mohanty P Kumar R Sharma R Puri V Singh PK. A study on the sentiments and psychology of twitter users during COVID-19 lockdown period. Multimed Tools Appl. (2022) 81:27009–31. doi: 10.1007/s11042-021-11004-w, PMID: 34149302 PMC8200552

[28] Singh C Imam T Wibowo S Grandhi S. A deep learning approach for sentiment analysis of COVID-19 reviews. Appl Sci. (2022) 12:3709. doi: 10.3390/app12083709

[29] Nezhad ZB Deihimi MA. Twitter sentiment analysis from Iran about COVID 19 vaccine. Diabetes Metab Syndr Clin Res Rev. (2022) 16:102367. doi: 10.1016/j.dsx.2021.102367, PMID: 34933273 PMC8667351

[30] Marcec R Likic R. Using twitter for sentiment analysis towards AstraZeneca/Oxford, Pfizer/BioNTech and Moderna COVID-19 vaccines. Postgrad Med J. (2022) 98:544–50. doi: 10.1136/postgradmedj-2021-140685, PMID: 34373343

[31] Niu Q Liu J Kato M Shinohara Y Matsumura N Aoyama T. Public opinion and sentiment before and at the beginning of COVID-19 vaccinations in Japan: twitter analysis. JMIR Infodemiol. (2022) 2:e32335. doi: 10.2196/32335, PMID: 35578643 PMC9092950

[32] Qorib M Oladunni T Denis M Ososanya E Cotae P. COVID-19 vaccine hesitancy: text mining, sentiment analysis and machine learning on COVID-19 vaccination twitter dataset. Expert Syst Appl. (2023) 212:118715. doi: 10.1016/j.eswa.2022.118715, PMID: 36092862 PMC9443617

[33] Chauhan U Shah A. Topic modeling using latent Dirichlet allocation. ACM Comput Surv. (2022) 54:1–35. doi: 10.1145/3462478

[34] Martínez NJF Pascual CP. Knowledge-based rules for the extraction of complex, fine-grained locative references from tweets. Revista Electron Linguist Aplicada. (2020) 19:136–63.

[35] Umakanthan S Sahu P Ranade AV Bukelo MM Rao JS Abrahao-Machado LF. Origin, transmission, diagnosis and management of coronavirus disease 2019 (COVID-19). Postgrad Med J. (2020) 96:753–8. doi: 10.1136/postgradmedj-2020-138234 32563999 PMC10016932

[36] Umakanthan S Patil S Subramaniam N Sharma R. COVID-19 vaccine hesitancy and resistance in India explored through a population-based longitudinal survey. Vaccine. (2021) 9:1064. doi: 10.3390/vaccines9101064, PMID: 34696172 PMC8537475

[37] Umakanthan S Senthil S John S Madhavan MK Das J Patil S. The effect of statins on clinical outcome among hospitalized patients with COVID-19: a multi-centric cohort study. Front Pharmacol. (2022) 13:1–11. doi: 10.3389/fphar.2022.742273, PMID: 35865966 PMC9294274

[38] Umakanthan S Lawrence S. Predictors of COVID-19 vaccine hesitancy in Germany: a cross-sectional, population-based study. Postgrad Med J. (2022) 98:756–64. doi: 10.1136/postgradmedj-2021-141365, PMID: 37062994

[39] Umakanthan S Bukelo MM Bukelo MJ Patil S Subramaniam N Sharma R. Social environmental predictors of COVID-19 vaccine hesitancy in India: a population-based survey. Vaccine. (2022) 10:1749. doi: 10.3390/vaccines10101749, PMID: 36298614 PMC9611416

[40] Umakanthan S Bukelo MM Gajula SS. The commonwealth Caribbean COVID-19: regions resilient pathway during pandemic. Front Public Health. (2022) 10:844333. doi: 10.3389/fpubh.2022.844333, PMID: 35664108 PMC9160791

[41] Nia ZM Ahmadi A Bragazzi NL Woldegerima WA Mellado B Wu J. A cross-country analysis of macroeconomic responses to COVID-19 pandemic using twitter sentiments. PLoS One. (2022) 17:e0272208. doi: 10.1371/journal.pone.0272208, PMID: 36001531 PMC9401163

[42] Reshi AA Rustam F Aljedaani W Shafi S Alhossan A Alrabiah Z. COVID-19 vaccination-related sentiments analysis: a case study using worldwide twitter dataset. Healthcare. (2022) 10:411. doi: 10.3390/healthcare10030411, PMID: 35326889 PMC8951387

[43] Anjorin AA Odetokun IA Abioye AI Elnadi H Umoren MV Damaris BF. Will Africans take COVID-19 vaccination? PLoS One. (2021) 16:e0260575. doi: 10.1371/journal.pone.0260575, PMID: 34851998 PMC8635331

[44] Kanyanda S Markhof Y Wollburg P Zezza A. Acceptance of COVID-19 vaccines in sub-Saharan Africa: evidence from six national phone surveys. BMJ Open. (2021) 11:e055159. doi: 10.1136/bmjopen-2021-055159, PMID: 34911723 PMC8678558

[45] Groves HE Piché-Renaud PP Peci A Farrar DS Buckrell S Bancej C. The impact of the COVID-19 pandemic on influenza, respiratory syncytial virus, and other seasonal respiratory virus circulation in Canada: a population- based study. Lancet Region Health Am. (2021) 1:100015. doi: 10.1016/j.lana.2021.100015, PMID: 34386788 PMC8285668

[46] Fontanet A Cauchemez S. COVID-19 herd immunity: where are we? Nat Rev Immunol. (2020) 20:583–4. doi: 10.1038/s41577-020-00451-5, PMID: 32908300 PMC7480627

[47] Solís Arce JS Warren SS Meriggi NF Scacco A McMurry N Voors M. COVID-19 vaccine acceptance and hesitancy in low-and middle-income countries. Nat Med. (2021) 27:1385–94. doi: 10.1038/s41591-021-01454-y, PMID: 34272499 PMC8363502

[48] Majeed B David JF Bragazzi NL McCarthy Z Grunnill MD Heffernan J. Mitigating co-circulation of seasonal influenza and COVID-19 pandemic in the presence of vaccination: a mathematical modeling approach. Front Public Health. (2023) 10:1–20. doi: 10.3389/fpubh.2022.1086849, PMID: 36684896 PMC9845909

[49] Soares P Rocha JV Moniz M Gama A Laires PA Pedro AR. Factors associated with COVID-19 vaccine hesitancy. Vaccine. (2021) 9:1–14. doi: 10.3390/vaccines9030300, PMID: 33810131 PMC8004673

[50] Silva MC Silva NCH Ferreira ALCG Ferreira FCG Melo MIB Silva LMX. Neutralizing antibodies against SARS-CoV-2 in Brazilian pregnant women vaccinated with one or two doses of BNT162b2 mRNA vaccine (Pfizer/WyethTM). Front Public Health. (2023) 10:1–7. doi: 10.3389/fpubh.2022.1054460, PMID: 36684877 PMC9845874

[51] Bussink-Voorend D Hautvast JL Vandeberg L Visser O Hulscher ME. A systematic literature review to clarify the concept of vaccine hesitancy. Nature human. Behaviour. (2022) 6:1634–48. doi: 10.1038/s41562-022-01431-6 35995837

[52] Shan D Xu J Liu T Zhang Y Dai Z Zheng Y. Subjective attitudes moderate the social connectedness in esports gaming during COVID-19 pandemic: a cross-sectional study. Front Public Health. (2022) 10:1–10. doi: 10.3389/fpubh.2022.1020114, PMID: 36684856 PMC9845587

[53] Xiong Y Weng X Snyder B Ma L Cong M Miller EL. Perceptions and knowledge regarding the COVID-19 pandemic between US and China: a mixed methods study. Glob Health. (2022) 18:1–14. doi: 10.1186/s12992-022-00864-y, PMID: 35941625 PMC9358088

[54] Takoudjou Dzomo GR Mbario E Djarma O Soumbatingar N Madengar M Djimera N. Predictors of COVID-19 vaccine hesitancy in Chad: a cross-sectional study. Front Public Health. (2023) 10:1–18. doi: 10.3389/fpubh.2022.1063954, PMID: 36684864 PMC9846328

[55] Caso D Carfora V Starace C Conner M. Key factors influencing Italian mothers’ intention to vaccinate sons against HPV: the influence of trust in health authorities, anticipated regret and past behaviour. Sustainability. (2019) 11:e6879. doi: 10.3390/su11236879

[56] Grodzicka ED. Taking vaccine regret and hesitancy seriously. The role of truth, conspiracy theories, gender relations and trust in the HPV immunisation programmes in Ireland. J Cult Res. (2021) 25:69–87. doi: 10.1080/14797585.2021.1886422

[57] Pokharel M Lillie HM Nagatsuka K Barbour JB Ratcliff CL Jensen JD. Social media narratives can influence vaccine intentions: the impact of depicting regret and character death. Comput Hum Behav. (2023) 141:e107612:107612. doi: 10.1016/j.chb.2022.107612

[58] Christy SM Winger JG Raffanello EW Halpern LF Danoff-Burg S Mosher CE. The role of anticipated regret and health beliefs in HPV vaccination intentions among young adults. J Behav Med. (2016) 39:429–40. doi: 10.1007/s10865-016-9716-z, PMID: 26782668 PMC4854757

[59] Penţa MA Crăciun IC Băban A. The power of anticipated regret: predictors of HPV vaccination and seasonal influenza vaccination acceptability among young Romanians. Vaccine. (2020) 38:1572–8. doi: 10.1016/j.vaccine.2019.11.042, PMID: 31786001

[60] Ziarnowski KL Brewer NT Weber B. Present choices, future outcomes: anticipated regret and HPV vaccination. Prev Med. (2009) 48:411–4. doi: 10.1016/j.ypmed.2008.10.006, PMID: 18996144

[61] Pan Y Ng CT Cheng TCE. Effect of free-riding behavior on vaccination coverage with customer regret. Comput Ind Eng. (2021) 159:e107494:107494. doi: 10.1016/j.cie.2021.107494

[62] Schwarzinger M Watson V Arwidson P Alla F Luchini S. COVID-19 vaccine hesitancy in a representative working-age population in France: a survey experiment based on vaccine characteristics. Lancet Public Health. (2021) 6:e210–21. doi: 10.1016/S2468-2667(21)00012-8, PMID: 33556325 PMC7864787

[63] Sallam M. COVID-19 vaccine hesitancy worldwide: a concise systematic review of vaccine acceptance rates. Vaccine. (2021) 9:160. doi: 10.3390/vaccines9020160, PMID: 33669441 PMC7920465

[64] Chung GKK Chan L Chan SM Chen JK Wong H Chung YN. The impact of trust in government on pandemic management on the compliance with voluntary COVID-19 vaccination policy among adolescents after social unrest in Hong Kong. Front Public Health. (2022) 10:1–12. doi: 10.3389/fpubh.2022.992895, PMID: 36660556 PMC9843607

[65] Solís Arce JS Warren SS Meriggi NF Scacco A McMurry N Voors M. COVID-19 vaccine acceptance and hesitancy in low- and middle-income countries. Nat Med. (2021) 27:1385–94. doi: 10.1038/s41591-021-01454-y, PMID: 34272499 PMC8363502

[66] Fu C Wei Z Pei S Li S Sun X Liu P. Acceptance of and preference for COVID-19 vaccination in healthcare workers: a comparative analysis and discrete choice experiment. MedRxiv. (2020) 4:1–35. doi: 10.1101/2020.04.09.20060103

[67] Maltezou HC Pavli A Dedoukou X Georgakopoulou T Raftopoulos V Drositis I. Determinants of intention to get vaccinated against COVID-19 among healthcare personnel in hospitals in Greece. Infect. Disease Health. (2021) 26:189–97. doi: 10.1016/j.idh.2021.03.002, PMID: 33906828 PMC8011642

[68] Lazarus JV Wyka K White TM Picchio CA Rabin K Ratzan SC. Revisiting COVID-19 vaccine hesitancy around the world using data from 23 countries in 2021. Nat Commun. (2022) 13:3801–14. doi: 10.1038/s41467-022-31441-x, PMID: 35778396 PMC9247969

[69] Biswas N Mustapha T Khubchandani J Price JH. The nature and extent of COVID-19 vaccination hesitancy in healthcare workers. J Community Health. (2021) 46:1244–51. doi: 10.1007/S10900-021-00984-3, PMID: 33877534 PMC8056370

[70] Heyerdahl LW Dielen S Nguyen T van Riet C Kattumana T Simas C. Doubt at the core: unspoken vaccine hesitancy among healthcare workers. Lancet Regional Health Europe. (2022) 12:100289–2. doi: 10.1016/j.lanepe.2021.100289, PMID: 34927116 PMC8668386

[71] Paget J Spreeuwenberg P Charu V Taylor RJ Iuliano AD Bresee J. Global mortality associated with seasonal influenza epidemics: new burden estimates and predictors from the GLaMOR project. J Glob Health. (2019) 9:9. doi: 10.7189/jogh.09.020421, PMID: 31673337 PMC6815659

[72] World Health Organization. Influenza (seasonal). World Health Organization (2022). Available online at: https://www.who.int/news-room/fact-sheets/detail/influenza-(seasonal) (Accessed February 10, 2023).

